# Imaging Study of Pseudo-CT Synthesized From Cone-Beam CT Based on 3D CycleGAN in Radiotherapy

**DOI:** 10.3389/fonc.2021.603844

**Published:** 2021-03-12

**Authors:** Hongfei Sun, Rongbo Fan, Chunying Li, Zhengda Lu, Kai Xie, Xinye Ni, Jianhua Yang

**Affiliations:** ^1^ School of Automation, Northwestern Polytechnical University, Xi’an, China; ^2^ Department of Radiotherapy, Second People’s Hospital of Changzhou, Nanjing Medical University, Changzhou, China; ^3^ Department of Radiotherapy, The Center of Medical Physics With Nanjing Medical University, Changzhou, China; ^4^ Department of Radiotherapy, The Key Laboratory of Medical Physics With Changzhou, Changzhou, China

**Keywords:** pseudo computed tomography (CT), CycleGAN, cone-beam computed tomography (CT), radiotherapy, cervical cancer

## Abstract

**Purpose:**

To propose a synthesis method of pseudo-CT (CT_CycleGAN_) images based on an improved 3D cycle generative adversarial network (CycleGAN) to solve the limitations of cone-beam CT (CBCT), which cannot be directly applied to the correction of radiotherapy plans.

**Methods:**

The improved U-Net with residual connection and attention gates was used as the generator, and the discriminator was a full convolutional neural network (FCN). The imaging quality of pseudo-CT images is improved by adding a 3D gradient loss function. Fivefold cross-validation was performed to validate our model. Each pseudo CT generated is compared against the real CT image (ground truth CT, CT_gt_) of the same patient based on mean absolute error (MAE) and structural similarity index (SSIM). The dice similarity coefficient (DSC) coefficient was used to evaluate the segmentation results of pseudo CT and real CT. 3D CycleGAN performance was compared to 2D CycleGAN based on normalized mutual information (NMI) and peak signal-to-noise ratio (PSNR) metrics between the pseudo-CT and CT_gt_ images. The dosimetric accuracy of pseudo-CT images was evaluated by gamma analysis.

**Results:**

The MAE metric values between the CT_CycleGAN_ and the real CT in fivefold cross-validation are 52.03 ± 4.26HU, 50.69 ± 5.25HU, 52.48 ± 4.42HU, 51.27 ± 4.56HU, and 51.65 ± 3.97HU, respectively, and the SSIM values are 0.87 ± 0.02, 0.86 ± 0.03, 0.85 ± 0.02, 0.85 ± 0.03, and 0.87 ± 0.03 respectively. The DSC values of the segmentation of bladder, cervix, rectum, and bone between CT_CycleGAN_ and real CT images are 91.58 ± 0.45, 88.14 ± 1.26, 87.23 ± 2.01, and 92.59 ± 0.33, respectively. Compared with 2D CycleGAN, the 3D CycleGAN based pseudo-CT image is closer to the real image, with NMI values of 0.90 ± 0.01 and PSNR values of 30.70 ± 0.78. The gamma pass rate of the dose distribution between CT_CycleGAN_ and CT_gt_ is 97.0% (2%/2 mm).

**Conclusion:**

The pseudo-CT images obtained based on the improved 3D CycleGAN have more accurate electronic density and anatomical structure.

## Introduction

Cervical cancer is one of the most common gynecological malignant tumors. According to statistics released in the 2017 annual meeting of the European Society for Medical Oncology (ESMO), the frequency of new cervical cancer cases was fourth highest in female cancers, and its fatality rate was third-highest (1). The main treatment means for cervical cancer are operation and comprehensive chemoradiotherapy (2). With the development of radiotherapy technology, image-guided radiation therapy (IGRT) has been gradually applied to clinical treatment of cervical cancer (3). In comparison with diagnostic CT, cone-beam CT (CBCT), a commonly used image-guided device, has higher spatial resolution, so it can be used for beam position verification of a patient between fractionated treatments (4). Rigid registration based on gray level or bone landmark is carried out through CBCT images scanned before each treatment and CT images acquired at the simulation stage (CT_sim_) to formulate a radiotherapy plan. Then, the setup error in 3D space can be determined to calibrate patient position (5). Given that the tumor target area of cervical cancer is closely related to the surrounding organs at risk (OARs) (such as the bladder and rectum), bladder filling and gastrointestinal peristalsis will directly affect the location of the tumor target area. The size and prescription dose of planning target volume (PTV) should be adjusted before each treatment to minimize the radiation dose of surrounding normal tissues. CBCT and standard multi-slice CT images are grayscale images that are processed and reconstructed by a computer after X-ray passes through different density tissues and organs, and the radiation energy after X-ray attenuation is measured by a flat panel detector. However, their imaging principles are different. CBCT uses 3D cone beam scanning instead of sector scan of multi-slice CT to obtain 2D projection data. Then, CBCT reconstructs the projection data obtained from different angles. Although the use of X-rays is improved, scattered signals are added, in turn causing the soft tissue resolution of CBCT images to decrease and produce more strip or band artifacts. The electron density is inaccurate and difficult to correct. Soft-tissue visualization also is hindered by tissue/breathing motion artifacts because of long (30–60 s) image acquisition. Therefore, CBCT images need to be modified to meet the requirements of clinical treatment (6).

Many correction methods are used for CBCT artifacts, including hardware-based pre-processing (7, 8) and software-based post-processing methods (9–12). Although these methods have been proven to be able to eliminate artifacts and improve image quality they cannot correct the HU values in CBCT images, and comprehensively considering the calculation amount and complexity of the algorithm, additional scanning time consumption, and incidental increased radiation dose, and clinical practicality is necessary. After consulting the literature, two main methods based on image post-processing are currently used for the correction of HU values in CBCT images. The first method is the image registration-based method, mainly including the deformation field registration and histogram matching methods. Chevillard et al. used an elastic deformation registration algorithm to establish the nonlinear mapping relation between CBCT and CT_sim_, and the CT image after registration not only had anatomical structure information of CBCT but also accurate electron density of CT_sim_ (13). Derksen et al. improved the deformation field registration method and constrained the deformation area by adding the OAR contours acquired by the image segmentation method, to improve registration accuracy between CBCT and CT_sim_ images (14). Abe et al. established the greyscale linear relation between CBCT and CT images by the histogram-matching method to correct Hounsfield unit (HU) values in CBCT images, and the experimental results showed that CBCT images after histogram matching could be applied to therapeutic plan formation of cervical and prostatic cancer (15). Although this type of method can correct HU information in the CBCT image, it has high accuracy requirements for the image registration algorithm and matching method, and the setting of an objective function is also complicated. The second method is the pseudo-CT synthesis-based method, which mainly includes machine learning and deep learning-based synthesis methods. Yang et al. proposed the alternate random forest method based on an automatic context model to extract multiscale texture features between CBCT and CT image pairs to establish nonlinear mapping relations and save the data model. New CBCT images were input into the trained model in the prediction phase to acquire virtual CT images with CBCT anatomical structure (16). Wang et al. used the fuzzy C-means clustering algorithm to classify voxel points inside CBCT images and assign CT values to voxel points according to weight information. Finally, they synthesized complete pseudo-CT images and verified the accuracy of HU values of pseudo-CT images from the aspect of dosimetry (17). Nevertheless, regardless of whether image registration or machine learning-based methods are used to synthesize pseudo-CT images, strict alignment of voxel information between CBCT and CT_sim_ images must be guaranteed and restricted by patient differences in bladder filling degree or soft tissue deformation in different periods of the scanning process. Acquiring CBCT and CT image pairs with completely matched anatomical structures is very difficult in practice.

To solve these problems, scholars have proposed the deep learning-based cycle generative adversarial network (CycleGAN) to synthesize pseudo images (18). Different from the traditional GAN network, CycleGAN is a loop network consisting of two GANs with mirror symmetry. The two GANs share two generators and two discriminators. This network is constrained by introducing a cycle consistency loss function to ensure that the model can effectively learn the nonlinear mapping relationship between unpaired image data in two image domains. Liang et al. applied the CycleGAN network to a pseudo image synthesis task between CBCT and CT_sim_ images and verified the accuracy of head and neck pseudo-CT images from the aspects of anatomical structure and dosimetry, respectively (19). Kida et al. proved that pseudo-CT images synthesized based on CycleGAN could be applied to prostate cancer treatment. Compared with the original CBCT, the image quality of the synthesized pseudo-CT image showed a substantial improvement in HU values, spatial uniformity, and artifact suppression. The anatomical structures of the CBCT image were well preserved in the synthesized image (20). However, these models are all applied to 2D CT image synthesis tasks. Spatial and structural information will be lost if a 2D convolutional kernel is used. Furthermore, the greater the number of 2D slices input into the network, the longer the model training time. In addition, due to artifacts caused by various factors in the CBCT image, the image quality will be degraded. Directly using the CycleGAN method to establish the mapping relationship between CBCT and CT images would result in falsely synthesized pseudo-CT images. Therefore, a 3D CycleGAN network carrying residual connection and attention gates was proposed in this study, and the gradient loss function was added into the objective function to further improve the accuracy of the synthesized CT images. The purpose of this study is to prove that electron density and organ relative position of the pseudo-CT images obtained based on improved CycleGAN are more accurate than those obtained by other deep learning methods. The accuracy of pseudo-CT images is also verified in terms of anatomy and dosimetry. The pseudo-CT images obtained by the new method are proved to have more accurate electron density for dose calculation. In this study, we also compared the pseudo-CT images obtained by 2D CycleGAN and 3D CycleGAN to prove the advantages of 3D network in the task of synthesizing pseudo-CT images. To avoid GPU memory restrictions imposed on 3D neural networks in training, the network was trained using a 3D image block-based network computing model, which could acquire abundant feature information while improving computing efficiency. On the basis of the literature review, this study is the first to use the 3D CycleGAN method to synthesize the 3D pseudo-CT from the CBCT image of the pelvic region.

## Materials and Methods

### Data Acquisition and Image Processing

A total of 120 sets of CT-CBCT image pairs used for training and prediction were obtained from 120 different patients. All image data selected in this experiment were 3D volume data of cervical cancer patients undergoing Volumetric Modulated Arc Therapy (VMAT). Among them, 100 cases were used for fivefold cross-validation to train the model, and the other 20 cases were used for testing. The CT images used in the entire process were obtained in the simulation stage, and the CBCT images were obtained by the patients after one week of treatment. CT images were acquired *via* an Optima CT520 device produced by GE Corporation (United States). Scanning conditions were as follows: tube voltage 120 kV, tube current 220 mA, image size 512×512×(102–119), and voxel spacing 0.9765×0.9765×3mm^3^. CBCT was equipped for an infinity linear accelerator, which was produced by Elekta Corporation (Sweden), was used to scan patients who had already accepted treatment for one week. Scanning conditions were as follows: tube voltage 120 kV, tube current 20 mA, image size 410×410×(50–76), and voxel spacing 1×1×5 mm^3^. CycleGAN input did not need two-group data with one-to-one registered voxel information. However, to improve the efficiency of operation, facilitate the training of the model, and reduce the interference of background voxel points outside the imaging area on image synthesis, we performed rigid registration based on bone landmarks on CT-CBCT image pairs to be input into the network. Before the model training, we also resampled the CT and CBCT images and preprocessed them with bicubic interpolation. Voxel spacing of preprocessed image data was unified as 1×1×1 mm^3^ and image size as 384×192×192. The minimum HU value of image data was unified as −1,000. For the convenience of GPU memory and acquiring refined image features, a complete 3D image was divided into 32×32×32 small image blocks in the experiment as input dimensions of the network model. During the acquisition of the image blocks, step size was set as 16, and an overlapping area occurred between adjacent image blocks, thereby ensuring that all imaging content was distributed in the image blocks and loss of image information was avoided.

### Pseudo-Computed Tomography Image Synthesis Based on 3D CycleGAN

**Figure 1:**
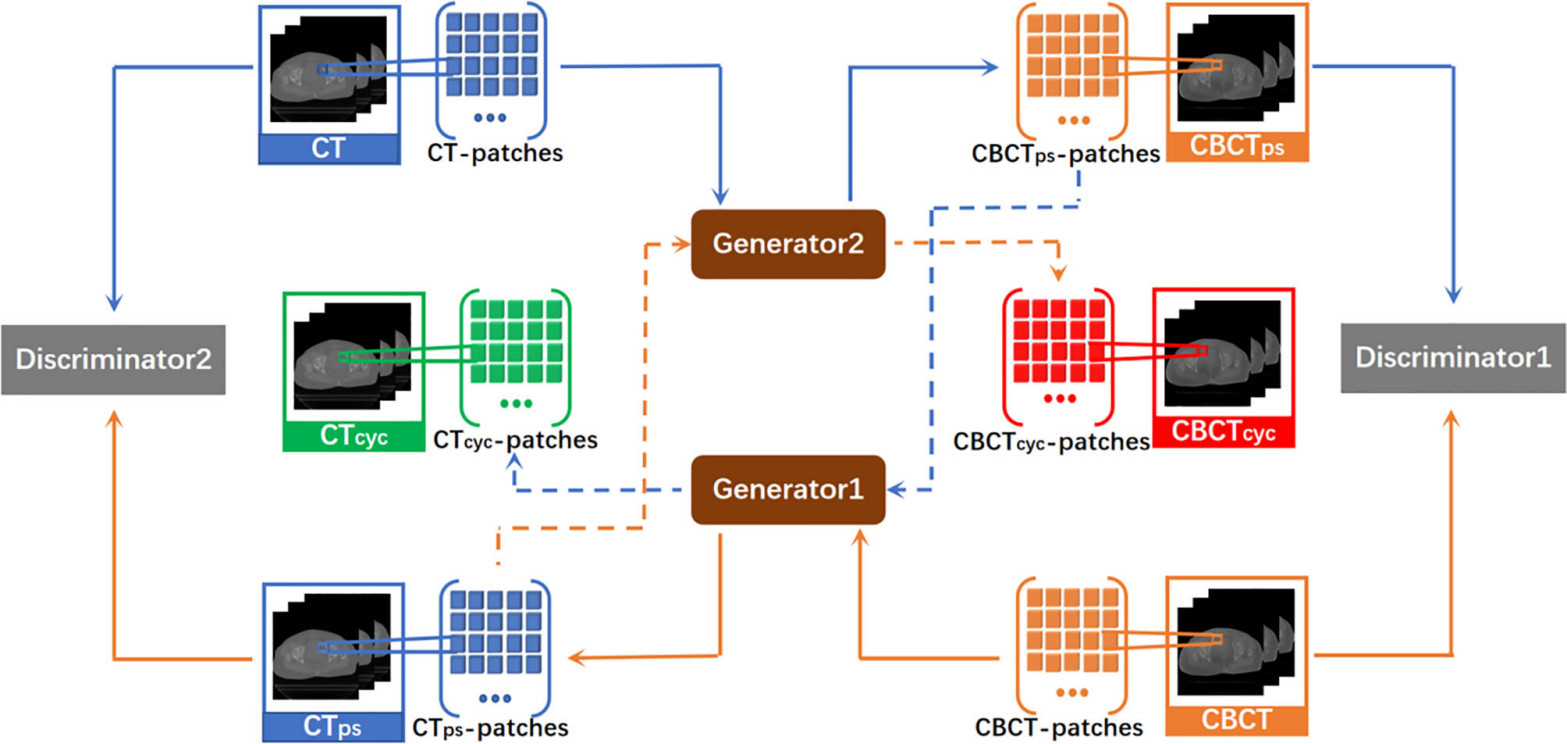
Total network structure of CycleGAN. It is a ring network composed of a GAN from CBCT to CT synthesis direction and a GAN from CT to CBCT synthesis direction. CT_ps_, pseudo CT obtained by generator1. CBCT_ps_, pseudo CBCT obtained by generator2. CT_cyc_, pseudo CT synthesized again through the cycle network. CBCT_cyc_, pseudo CBCT synthesized again through the cycle network.

The traditional GAN is unidirectional. CycleGAN used in this experiment was a loop network consisting of one unidirectional CBCT→CT GAN and another unidirectional CT→CBCT GAN. The CycleGAN contained two discriminators, in which discriminators 1 and 2 were used to judge the authenticity of the CBCT and CT images, respectively. CycleGAN also included two generators that were each used to generate pseudo-CT and pseudo-CBCT images. By acquiring an input image from original domain A, this model transmitted the input image to the first generator in the form of voxel block, converted it into an image block in target domain B, and reconstructed a complete image. The generated image was also used as the input in the form of voxel block to be transmitted into the second generator, converted into an image block in original domain A, and reconstructed into an output image. This output image must be approximate to the original input image in gray level and anatomical structure. Here, the nonlinear mapping relationship between two unpaired image data is set. CBCT and CT images served as input images of the original domain to train two independent GAN networks. An association was established through the cycle consistency loss function to constitute a complete CycleGAN. Its overall network structure is shown in **Figure 1**.

### Generators and Discriminators of 3D CycleGAN

**Figure 2:**
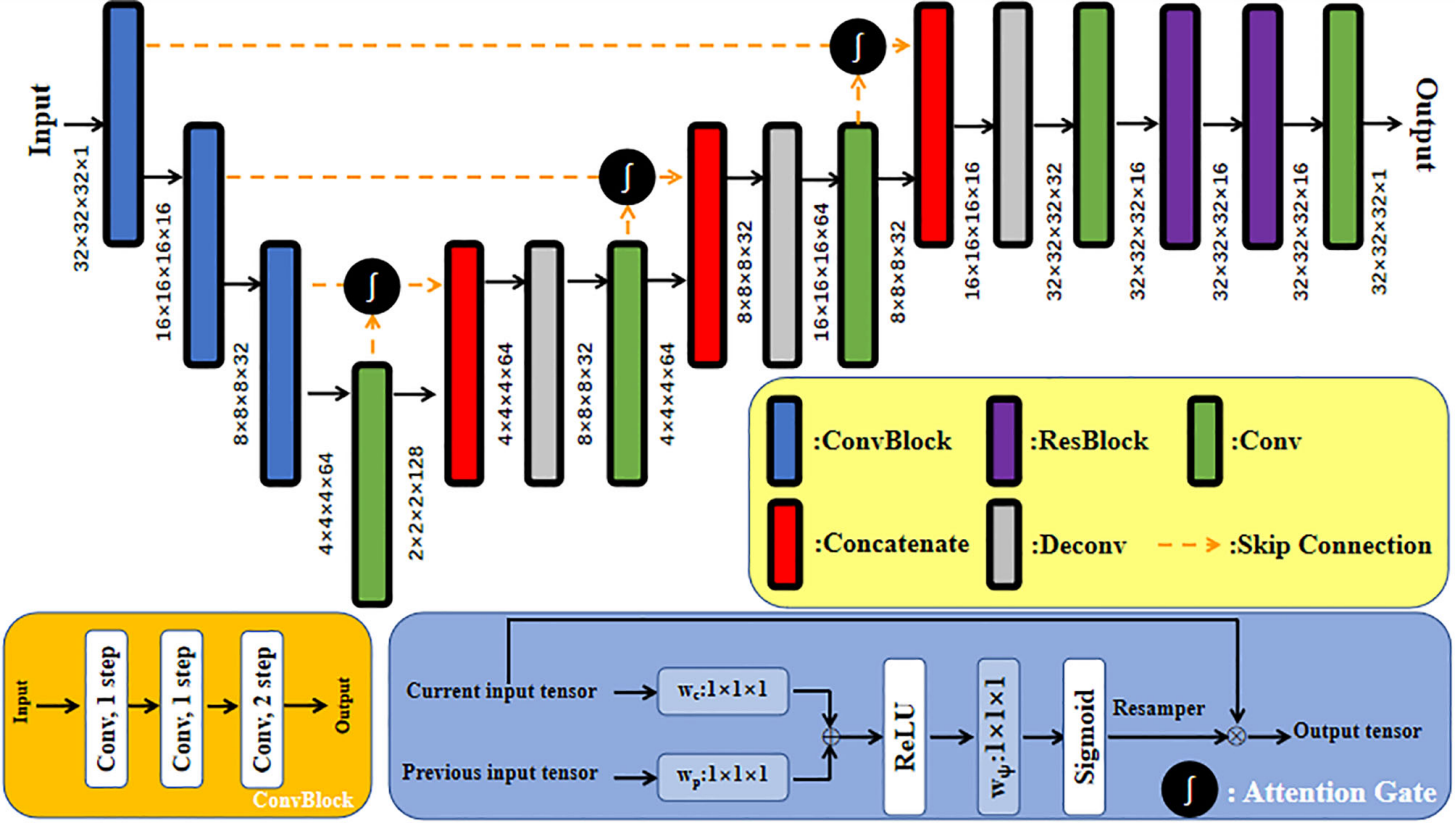
U-Net network structure of generator with residual connection and attention gates.

A CNN with a residual connection has already been proven to have excellent application effects in many image processing tasks (21–23). This CNN expresses network output as a linear superposition of nonlinear transformation of inputs through identity shortcut connection. In comparison with a directly connected convolutional neural network, ResNet directly transmits feature information of the input network along the shortcut, which can protect information integrity to a certain degree, simplifying and clarifying the model learning goal and solving the gradient missing problem in training (24). In addition, the attention gate has been proven to be able to complete the CBCT→CT image synthesis task well. The model with attention gates uses attention coefficients to highlight image regions with salient features and suppress the feature responses of irrelevant regions during training, that is, effectively suppress the artifact regions in CBCT images. Interested readers can refer to the paper by Liu et al. (25) on the detailed design of the network with an attention gate. The generator used in this study was a deep convolutional neural network similar to U-Net with residual connection and attention gates, in which 32×32×32 patch voxel blocks in CT_sim_ and CBCT image domains were used as inputs of the synthesis direction of the two pseudo images in the network. Before each skip connection, the network with attention gates added a gating signal to the output of the encoder and corresponding decoder under each resolution. These signals were used to define the importance of image features at different positions in 3D image space and readjust the output features of the network layer. The patch blocks input into the generative network first passed through three ConvBlock blocks. ConvBlock consisted of two convolutional layers with a step size of 1 and one convolutional layer with a step size of 2, in which each convolutional layer included conv, BN, and LReLU operations, and the padding was SAME. The output abstract features passed through another three-group concatenate and deconvolutional layers, and the inputs of each concatenate group were the output of the previous convolutional layer and the output after it passed through the attention gate module together with its corresponding ConvBlock. Each deconvolutional layer included deconv, BN, and LReLU operations. Step size was 2 and the padding was SAME. Abstract features passed through the remaining ResNet and convolutional layers of the generator, aiming to enhance network nonlinearity. The dimensions of the convolution kernel used in all network layers were 3×3×3. The concrete network structure of the generator is shown in **Figure 2**.

**Figure 3:**
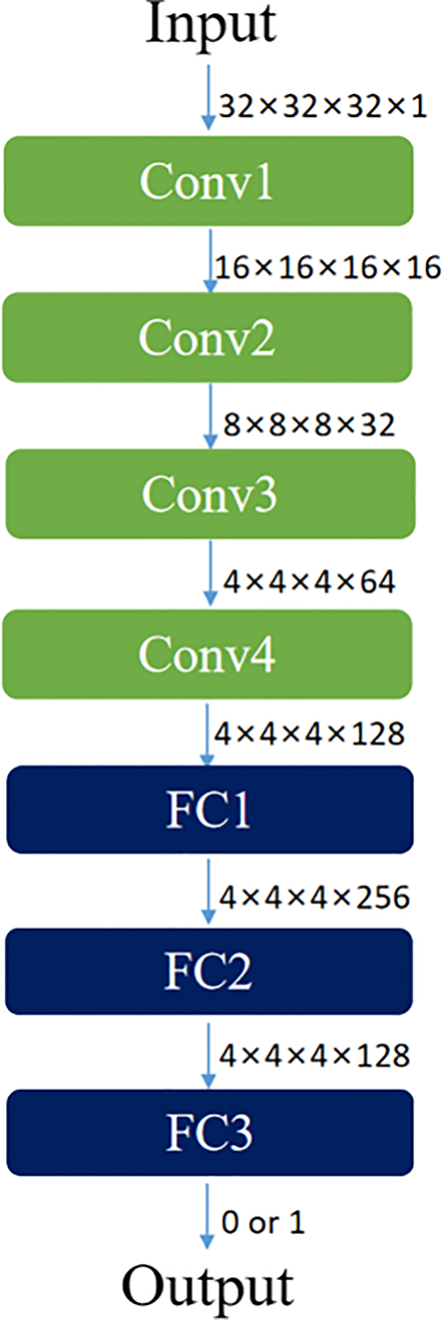
Network structure diagram of discriminator based on FCN.

Both discriminators of CycleGAN were conventional full convolutional neural networks (FCN), which received patch blocks in the CBCT and CT image domains as inputs, respectively. Each discriminator contained four convolution layers and three fully connected layers. Each convolutional layer included convolution, BN, and LReLU operations. Dimensions of convolution kernel were 4×4×4; step sizes were 2, 2, 2, and 1, respectively; and padding was SAME. LReLU served as the activation function in the first two fully connected layers, and the Sigmoid activation function was used in the third fully connected layer to acquire judgment results of the discriminator regarding the authenticity of the input image. The result value was a probability. Feature maps at all layers of the discriminator were 16, 32, 64, 128, 256, 128, and 1, respectively. The concrete network structure of the discriminator is shown in **Figure 3**.

### Loss Functions of 3D CycleGAN

The loss function of the CycleGAN network contains two parts: generator loss and discriminator loss functions. The main task of the discriminator is to distinguish real image data from pseudo image data synthesized *via* a generator. According to the GAN network structure of mirror symmetry, CycleGAN has discriminators in two image domains, where discriminator 1 is used to judge the authenticity of CBCT data. To calculate the loss function, discriminator 1 includes two inputs and two corresponding outputs. Discriminator 1 uses pseudo-CBCT image G_2_(X_CT_) generated by generator 2 as an input to obtain output D(G_2_(X_CT_)) and real CBCT image Y_CBCT_ as another input to obtain output D(Y_CBCT_). Hence, the loss function of discriminator 1 is defined as follows:

(1) $$L_{D_{1}}=L_{BCE}(1,D(Y_{CBCT}))+L_{BCE}(0,D(G(X_{CT})))$$

where L_BCE_ is the binary cross-entropy loss function, which is defined in Formula 2. Z represents an input image data label, the value of which is taken as 1 or 0 based on data authenticity. Z^’^ denotes the probability for the discriminator to predict the input image as a real or pseudo image, and its value range is [0,1].

(2) $$L_{BCE}(Z,Z^{'})=-\sum_{i=1}^{N}Z_{i}\log(Z_{i}^{'})+(1-Z_{i})\log(1-Z_{i}^{'})$$

Discriminator 2 is used to judge the authenticity of CT data. It also includes two inputs and two outputs, and its loss function is defined as follows:

(3) $$L_{D_{2}}=L_{BCE}(1,D(Y_{CT}))+L_{BCE}(0,D(G(X_{CBCT})))$$

where Y_CT_ is a real CT image, and the corresponding output is D(Y_CT_). G(X_CBCT_) is a pseudo image generated by generator 1. The corresponding output is D(G(X_CBCT_)), and the total loss function of this discriminator is shown in Formula 4.

(4) $$L_{D}=L_{D}1+L_{D_{2}}$$

The main task of the generator is to acquire pseudo image data which is as approximate as possible to real input image data in the aspects of gray level and anatomical structure, to perplex the generator. The loss function of the generator includes adversarial, cycle consistency, and gradient losses. For generator 1, its adversarial loss is the binary cross-entropy L_BCE_ (1, D (G_1_(X_CBCT_))) of the probability for discriminator 2 to discriminate pseudo-CT image G_1_(X_CBCT_) generated by generator 1 as a real image. The probability is 1. Similarly, the loss function of generator 2 is binary cross-entropy L_BCE_ (1, D(G_2_(X_CT_))) of probability D(G_2_(X_CT_)) and 1.

In addition to the adversarial loss of classical GAN, the CycleGAN network also has cycle loss (26). The network needs to ensure that the generated image reserves the characteristics of the original image. Thus, if one generator in the network is used to generate a 3D pseudo image, then the other generator should be used to recover the original input 3D image data as much as possible. This process needs to satisfy cycle consistency. L1 loss was used in this study as cycle consistency loss. Cycle consistency losses of generators 1 and 2 are defined as follows:

(5) $$L_{cyc-G_{1}}=\sum_{i=1}^{N}||G_{2}(G_{1}(X_{CBCT}))-X_{CBCT}|{|}_{2}$$

(6) $$L_{cyc-G_{2}}=\sum_{i=1}^{N}||G_{1}(G_{2}(X_{CT}))-X_{CT}|{|}_{2}$$

In addition, the L1 loss function used in the cycle consistency loss will lead to image fuzziness, a gradient loss function was added in this study to enhance 3D gradient similarity between pseudo image data synthesized by the generator and real image data so that the texture information of the pseudo image can be as accurate as possible. Gradient loss functions L_GL-CT_ and L_GL-CBCT_ are defined in Formulas (7) and (8), respectively.

(7) $$L_{GL-CT}={\left|\left|\nabla G_{1}{\left(X_{CBCT}\right)}_{x}|-|\nabla X_{CT}x\right|\right|}^{2}+{\left|\left|\nabla G_{1}{\left(X_{CBCT}\right)}_{y}|-|\nabla X_{CT}y\right|\right|}^{2}+{\left|\left|\nabla G_{1}{\left(X_{CBCT}\right)}_{z}|-|\nabla X_{CT}z\right|\right|}^{2}$$

(8) $$L_{GL-CBCT}={\left|\left|\nabla G_{2}{\left(X_{CT}\right)}_{x}|-|\nabla X_{CBCT}x\right|\right|}^{2}+{\left|\left|\nabla G_{2}{\left(X_{CT}\right)}_{y}|-|\nabla X_{CBCT}y\right|\right|}^{2}+{\left|\left|\nabla G_{2}{\left(X_{CT}\right)}_{z}|-|\nabla X_{CBCT}z\right|\right|}^{2}$$

In summary, the total loss function of the generator is as follows:

(9) $$L_{G}=L_{BCE}(1,D(G_{1}(X_{CBCT})))+L_{BCE}(1,D(G_{2}(X_{CT})))+\lambda_{1}L_{Cyc-G_{1}}+\lambda_{2}L_{Cyc-G_{2}}+\lambda_{3}L_{GL-CT}+\lambda_{4}L_{GL-CBCT}$$

where λ_1_=λ_2 =_ 10 and λ_3_=λ_4 =_ 0.5.

### Cross-Validation of the Trained Model

To validate the model’s performance, a fivefold cross-validation technique was used for training and testing steps, where 100 cases are randomly partitioned into five groups. For each experiment, four groups (including 80 cases) are selected for testing the trained model. Once the model is trained, it is applied to each test subject’s CBCT image to generate the pseudo CT. Pseudo CT synthesized based on a 3D GAN with a U-Net generator (CT_unet-GAN_) and 3D GAN with an FCN generator (CT_FCN-GAN_) were selected as the control experiments to verify the accuracy of the pseudo-CT images acquired based on the improved CycleGAN (27, 28).

The accuracy of each subject’s pseudo CT and real CT was evaluated using the voxel-wise mean absolute error (MAE) calculated in the pelvic region:

(10) $$MAE(CT_{real},CT_{ps})=\frac{1}{N}\sum_{i=1}^{N}\left|CT_{real}(i)-CT_{ps}(i)\right|$$

where the N is the total number of the voxels in the pelvic region of the CT. The CT_real_ is the real image scanned by a CT machine. The CT_ps_ is the pseudo CT obtained based on the improved CycleGAN. Another metric used to evaluate the prediction accuracy of the model is the structural similarity coefficient (SSIM). Its mathematical definition is as follows:

(11) $$SSIM=\frac{\left(2\mu_{r}\mu_{p}+C_{1})(2\delta_{rp}+C_{2}\right)}{\left(\mu_{r}^{2}+\mu_{p}^{2}+C_{1})(\delta_{r}^{2}+\delta_{p}^{2}+C_{2}\right)}$$

*µ_r_* and *µ_p_* are the mean values of HU of real CT image and pseudo-CT image, respectively, *δ_r_* and *δ_p_* is the variance of HU values of real CT image and pseudo-CT image, respectively, *δ_rp_* is the covariance, the parameters *C*_1_ = (*k*_1_ *L*)^2^ and *C*_2_ = (*k*_2_ *L*)^2^ are two variables to stabilize the division with weak denominators, L is the range of HU values in CT image. k_1 =_ 0.01, k_2 =_ 0.02. The SSIM value range is [0,1], the closer the value is to 1, the greater the similarity between the two images.

### Evaluation

Dice similarity coefficient (DSC) (29) was used to evaluate the accuracy difference between pseudo-CT images obtained by different methods and CT_gt_ images on multiple organs at risk. In this study, the distinct curve-guided FCN proposed by He et al. was used to segment the OARs in the pelvic region of the pseudo-CT images and CBCT images (30). Segmentation accuracies of bladder and uterus regions in the pseudo-CT images were evaluated through DSC. The ground truth is the contour of the bladder, uterus, rectum, and bone regions manually segmented on the CT_gt_ images. The overlapping ratio of OARs between pseudo-CT images obtained through different algorithms and CT_gt_ images was calculated. An accurate segmentation result should have a high overlapping ratio of organ volumes. DSC is defined as follows:

(12) $$DSC=\frac{2|L_{CT_{gt}}\cap L_{CT_{ps}}|}{\left|L_{CT_{gt}}|+|L_{CT_{ps}}\right|}$$

where $L_{CT_{gt}}$ and $L_{CT_{ps}}$ represent segmentation results of OARs in real CT and pseudo-CT images acquired through different algorithms, respectively. The closer the DSC value to 1, the higher the similarity between OAR regions in pseudo-CT image and the corresponding region in CT_gt_ image.

Two quantitative measurement methods, namely, normalized mutual information (NMI) (31), peak signal-to-noise ratio (PSNR) (32), and were used in this study to evaluate the accuracy of pseudo-CT images obtained through 3D and 2D CycleGAN in anatomical structure.

The first quantitative index is NMI, which is used to evaluate the similarity between pseudo-CT images acquired through different methods and CT_gt_. Its expression is as follows:

(13) $$NMI(CT_{gt},CT_{ps})=\frac{2I(CT_{gt},CT_{ps})}{H(CT_{gt})+H(CT_{ps})}$$

(14) $$I(CT_{gt},CT_{ps})=H(CT_{gt})-H(CT_{gt}|CT_{ps})=H(CT_{gt})-H(CT_{ps}|CT_{gt})$$

I(CT_gt_,CT_ps_) is the mutual information value between pseudo-CT and ground truth CT images. H(CT_gt_) and H(CT_ps_) are information entropies. The closer the NMI value is to 1, the better the image registration effect.

The second quantitative index is PSNR, the formula of which is as follows:

(15) $$PSNR\backslash !=\backslash !20\log_{10}\backslash !(\backslash !\frac{MAX_{I}}{\sqrt{\frac{1}{XYZ}\sum_{x=0}^{X-1}\sum_{y=0}^{Y-1}\sum_{z-1}^{Z-1}|I_{gt}(x,y,z)\backslash !-\backslash !I_{ps}(x,y,z){|}^{2}}}\backslash !)$$

In Formula (13), *I_gt_* and *I_ps_* denote CT_gt_ and pseudo-CT images, respectively. X, Y, and Z represent image sizes. *MAX_I_* is the maximum gray value in the CT image. The greater the PSNR value, the more approximate the synthesized pseudo-CT image to the CT_gt_ image.

Pseudo-CT images synthesized through different deep learning methods and CT images acquired through registration were imported into the Monaco planning system (Elekta, Sweden), where the latter was selected as the ground truth image for dosimetry verification. Three radiotherapists with rich clinical experience jointly re-delineated PTVs and OARs on a CT_gt_ image and copied them onto pseudo-CT and CBCT images. VMAT radiotherapy plans were prepared respectively on CT_gt_ and pseudo-CT images acquired through three deep learning methods based on Monte Carlo algorithm. The dose of the original 4500 cGy/25 F prescription was modified into the new prescription dose 3600 cGy/20 F. Dose calculation was implemented *via* the Monte Carlo algorithm based on the CT_gt_ image, and then the optimized plan was copied onto different pseudo-CT images and CBCT image after conforming to clinical requirements. To compare the difference between pseudo-CT and CT_gt_ images in the radiotherapy plan, the 3600 cGy prescription dose with 95% PTV was used as the passing criterion of the plan. The doses in PTV and OARs of cervical cancer patients, which were obtained based on pseudo-CT and CT_gt_ images under the same optimization conditions of VMAT treatment in the planning system, were compared. OARs included bladder, femoral head, and small intestine. The main dosimetry evaluation indexes included dose-volume histogram (DVH), dose covering 98% of the PTV (D98%), mean dose (Dmean), and dose to 2% of the PTV (D2%). In addition, based on the dose distribution of CT_gt_, the pass rate of γ analysis was evaluated for the central level dose of pseudo CT obtained by three methods (33). The parameter standard was 2%/2 mm (dose difference 2%, distance difference 2 mm).

## Results

### Evaluation of Anatomical Structure

As for anatomical structure verification, **Table 1** provides a summary of MAE and SSIM metrics computed based on the real and different pseudo CT for each fold in the fivefold validation. Compared with other GAN methods, the pseudo-CT synthesized by the improved CycleGAN method proposed in this study has higher accuracy, and its MAE value decreases and SSIM value increases. This finding indicates that the results obtained by CycleGAN with gradient information in the unpaired CBCT and CT image synthesis tasks are closer to the real CT images with higher quality.

**Table 1:** Metric results of MAE and SSIM computed between real CT image and different pseudo-CT images for fivefold cross-validation.

	Metric	Fold 1	Fold 2	Fold 3	Fold 4	Fold 5
**CycleGAN**	MAE	52.03 ± 4.26HU	50.69 ± 5.25HU	52.48 ± 4.42HU	51.27 ± 4.56HU	51.65 ± 3.97HU
	SSIM	0.87 ± 0.02	0.86 ± 0.03	0.85 ± 0.02	0.85 ± 0.03	0.87 ± 0.03
**Unet-GAN**	MAE	55.36 ± 5.42HU	56.33 ± 5.02HU	57.18 ± 5.23HU	57.22 ± 5.06HU	56.54 ± 5.58HU
	SSIM	0.80 ± 0.03	0.82 ± 0.03	0.82 ± 0.02	0.81 ± 0.02	0.81 ± 0.02
**FCN-GAN**	MAE	67.63 ± 5.49HU	65.36 ± 6.02HU	62.34 ± 6.35HU	62.97 ± 7.19HU	63.64 ± 7.87HU
	SSIM	0.75 ± 0.04	0.74 ± 0.03	0.78 ± 0.02	0.79 ± 0.02	0.77 ± 0.03

**Figure 4:**
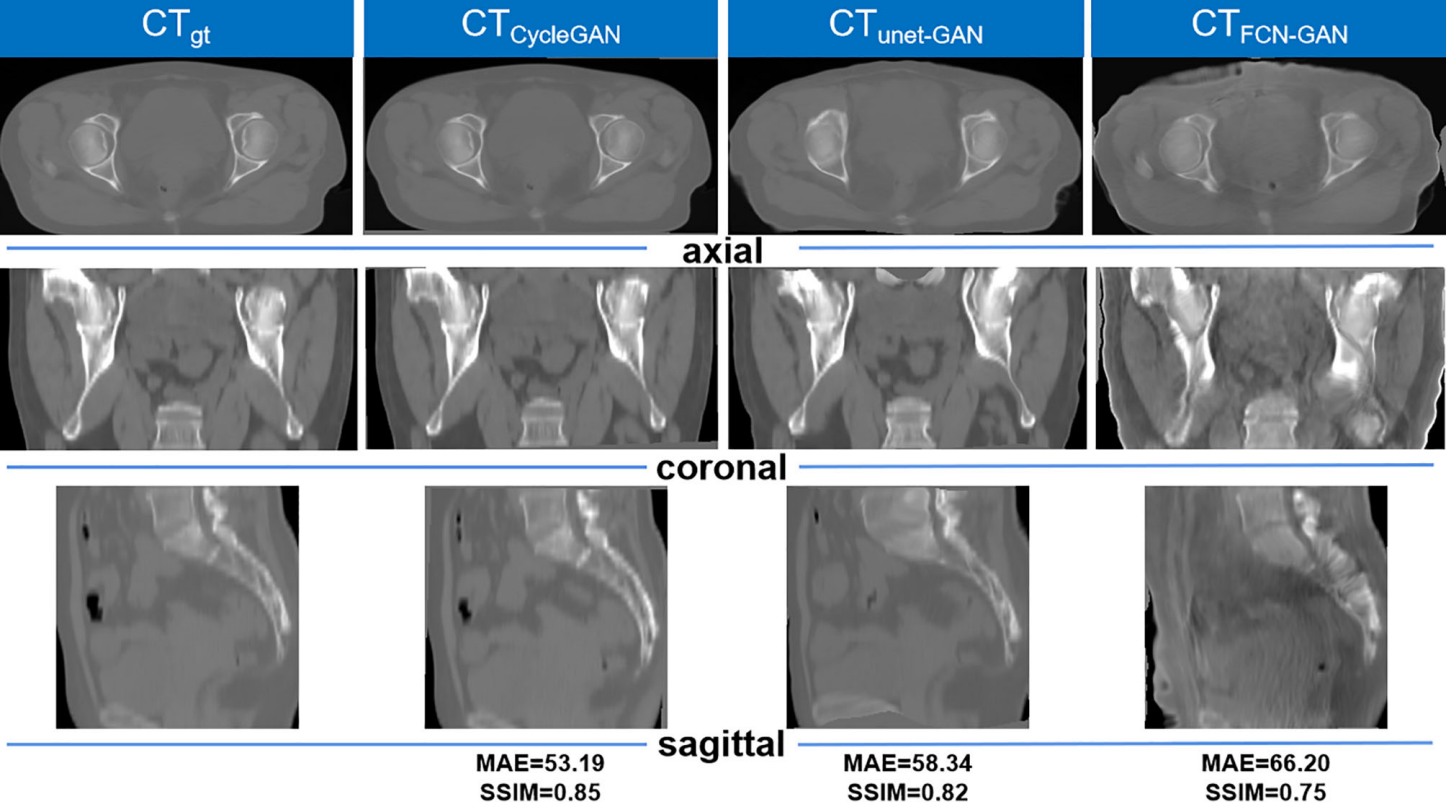
Comparison of the results of CT_gt_ images and pseudo-CT images obtained based on different deep learning methods in the axial, coronal, and sagittal directions. CT_gt_: The real CT images. CT_CycleGAN_: Pseudo CT obtained based on CycleGAN. CT_unet-GAN_: Pseudo CT obtained by GAN with U-net generator. CT_FCN-GAN_: Pseudo CT obtained by GAN with FCN generator. MAE: mean absolute error. SSIM: structural similarity coefficient.

**Figure 5:**
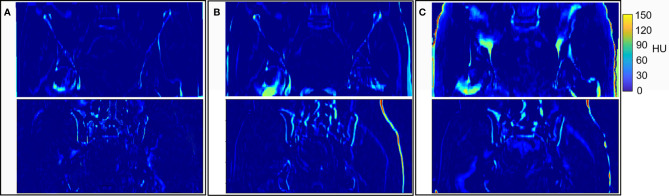
Results of HU values difference between CT_gt_ and pseudo CT obtained by different deep learning methods. **(A)** HU values difference between CT_gt_ and CT_CycleGAN_. **(B)** HU values difference between CT_gt_ and CT_unet-GAN_. **(C)** HU values difference between CT_gt_ and CT_FCN-GAN_.

The graphical results of CT_gt_ image and pseudo-CT images acquired through different deep learning methods in three axial directions are presented in **Figure 4**. Here, CT_gt_ was a CT image after registration of CBCT and CT_sim_ images. The specific registration method has been clarified in the II. D.section. Given that the 3D FCN-GAN method could largely acquire pseudo-CT images (CT_FCN-GAN_), but its imaging quality was poor, the resolution of soft tissues was low and the bone region underwent deformations to different degrees. In comparison with the former, a pseudo-CT image (CT_unet-GAN_) acquired based on the 3D Unet-GAN method had a better effect, but the skeleton region still experienced partial deformation and some soft tissues were inaccurate. The pseudo-CT image (CT_CycleGAN_) acquired based on the 3D CycleGAN method was the most approximate to CT_gt_ image in anatomical structure, and textures of soft tissues and organs in this image were similar to those in the CT_gt_ image. **Figure 5** shows CT value difference plots between pseudo-CT images acquired through different deep learning methods and CT_gt_, where 5(a) shows the CT value difference plot between CT_gt_ and CT_CycleGAN._ Their CT value difference in the soft tissue region was within 50 HU. **Figure 5B** displays the CT value difference plot between CT_gt_ and CT_unet-GAN_, and 5(c) is that between CT_gt_ and CT_FCN-GAN_. CT values of the latter two pseudo-CT images were different from those of CT_gt_ in the skeleton and soft tissue regions to different degrees. **Table 2** presents the DSC measurement results of 3D volume overlapping differences of ladder, uterus, rectum, and bone regions between real CT and different pseudo-CT images of predicted volume data of 20 cases. Different OARs in CT_CycleGAN_ and CT_gt_ images had higher DSC values.

**Table 2:** DSC (%) measurement results of segmentation accuracy between real CT images and pseudo-CT images synthesized by three different methods.

Organ	Method		
	CycleGAN	Unet-GAN	FCN-GAN
**bladder**	91.58 ± 0.45*	88.63 ± 0.51	87.83 ± 0.56
**uterus**	88.14 ± 1.26*	87.52 ± 1.60	85.31 ± 2.72
**rectum**	87.23 ± 2.01*	85.64 ± 2.33	84.39 ± 3.51
**bone**	92.59 ± 0.33*	89.57 ± 0.47	86.78 ± 0.58

*Indicates significant improvement via paired t -tests (p < 0.05).

The bold is used to show the best performance.

**Figure 6:**
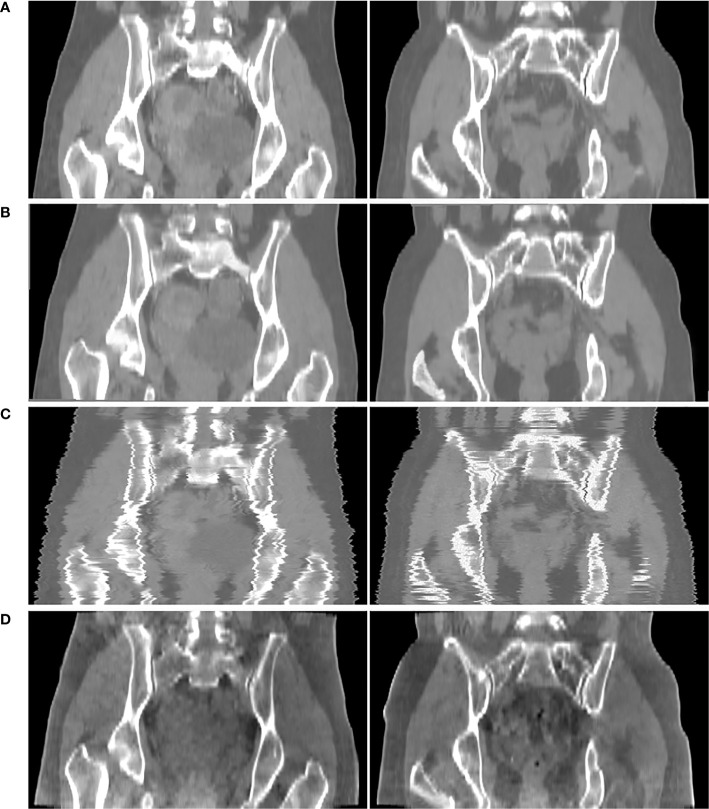
Comparison results of CT_gt_ and pseudo CT obtained based on 3D and 2D CycleGAN. **(A)** real CT images(CT_gt_). **(B)** pseudo-CT images obtained based on 3D CycleGAN. **(C)** pseudo-CT images obtained based on 2D CycleGAN. **(D)** CBCT images.

**Figure 6** shows the comparison result of pseudo CT obtained based on 3D and 2D CycleGAN. The pseudo-CT images synthesized by 2D CycleGAN are the result of training after modifying the network layer of the 3D network and the loss function to the 2D mode. **Figure 6A** is the real CT images. **Figures 6B, C** show the 3D pseudo-CT images based on 3D CycleGAN and 2D CycleGAN with interpolation reconstruction. **Figure 6D** shows the CBCT images. Compared with the 2D results, the organ structures in the pseudo-CT images are more continuous in the Z direction. **Table 3** shows the evaluation results between pseudo-CT and real CT images based on 3D and 2D CycleGAN under NMI and PSNR measurement methods, and the comparison results between CBCT and real CT images are used as a reference. The numerical results indicate that, compared with CBCT images, the HU values in pseudo-CT images obtained by the two CycleGAN methods are closer to the real CT images, but the 3D method is more accurate than the 2D method.

**Table 3:** Metric results between real CT images and different CT images of 20 patients with cervical cancer.

Metric	CT images		
	CT_CycleGAN-3D_	CT_CycleGAN-2D_	CBCT
NMI	0.90 ± 0.01	0.87 ± 0.02	0.79 ± 0.02
PSNR (dB)	30.70 ± 0.78	29.72 ± 0.59	27.15 ± 0.57

CT_CycleGAN-3D_, pseudo-CT images obtained based on 3D CycleGAN; CT_CycleGAN-2D_, pseudo-CT images obtained based on 2D CycleGAN.

**Figure 7:**
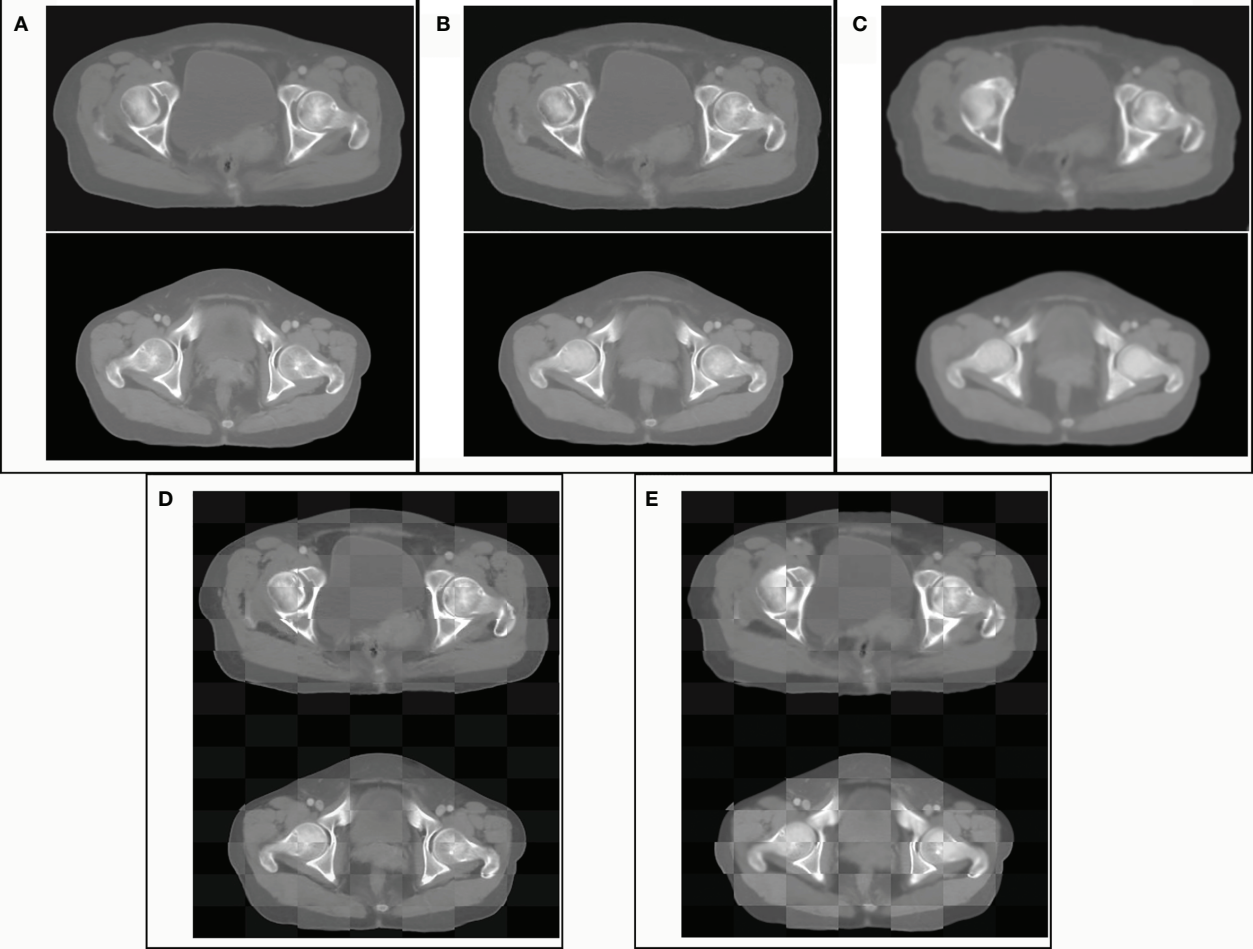
Results of pseudo-CT images obtained by adding gradient loss function and not adding it in CycleGAN. **(A)** CT_gt._ **(B)** pseudo CT obtained by CycleGAN with gradient loss. **(C)** pseudo CT obtained based on CycleGAN without gradient loss. **(D)** The comparison result of **(A, B)**. **(E)** Comparison result of **(A, C)**.

In addition, to prove the training effect of the gradient loss function on CycleGAN, we use CycleGAN with gradient loss and CycleGAN without it to perform pseudo-CT synthesis. The result is shown in **Figure 7**. For the CycleGAN method without gradient loss, the synthetic pseudo-CT image is generally fuzzy, caused by the L2 Euclidean distance loss function in the cyclic consistency loss function. In terms of details, the difference in areas with large gray gradient changes such as bones is more obvious, the edge information between organs is blurred, and the overall skin contour of patients is not accurate. **Figures 7D, E** also show that the improved CycleGAN with gradient loss can obtain pseudo CT with more accurate anatomical structure. In **Figures 7D, E**, the area with a bright visual effect is the CT_gt_ with a window range of (−600,600)HU, whereas the area with a dark visual effect is the pseudo CT with a window range of (−400,800)HU.

### Dosimetric Evaluation

**Figure 8:**
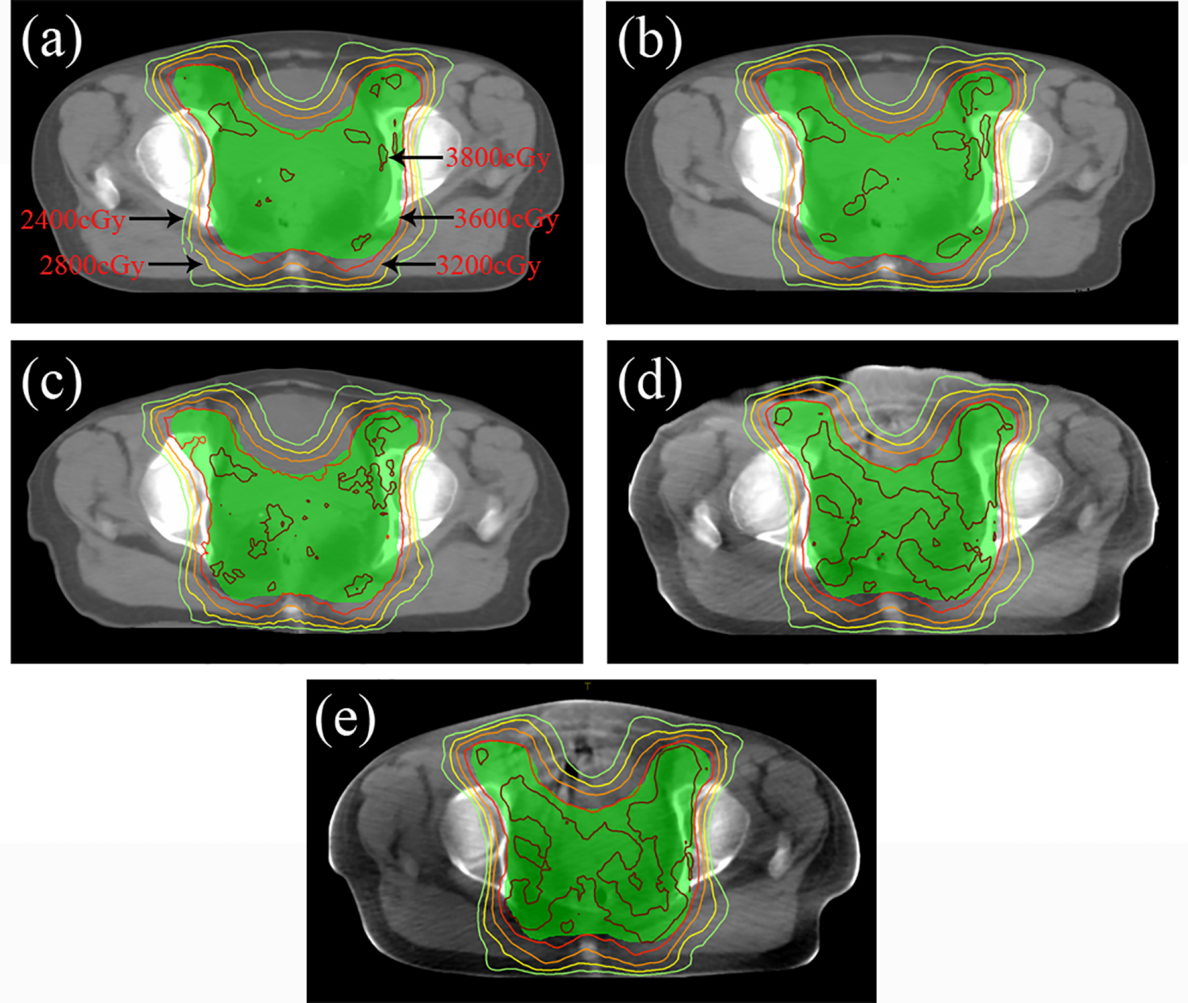
Dose distribution of radiotherapy plan based on four CT images at the isocenter level. **(A)** Dose distribution of CT_gt_ at isocenter. **(B)** Dose distribution of CT_CycleGAN_ at isocenter. **(C)** Dose distribution of CT_unet-GAN_ at isocenter. **(D)** Dose distribution of CT_FCN-GAN_ at isocenter. **(E)** Dose distribution of CBCT at isocenter.

In terms of dosimetry verification, the cross-sectional dose distributions of the treatment plan casting on CT_gt_, CT_CycleGAN_, CT_unet-GAN_, CT_FCN-GAN_, and CBCT images for one of the testing patients were shown in **Figures 8A–E**. The experimental results showed that the dose distribution difference between CT_CycleGAN_ and CT_gt_ in overall PTV was minor, and the dose distribution in the high-dose region of CT_CycleGAN_ was approximate to that of CT_gt_. PTV is a region that includes part of the uterus, bladder, rectum, and other OARs. PTV is delineated with reference to the RTOG 63 report. The dose in the intersection region between the femoral head and PTV of CT_unet-GAN_ was deficient with inaccurate dose distribution. Many high-dose regions were found in PTV of CT_FCN-GAN_.

**Figure 9:**
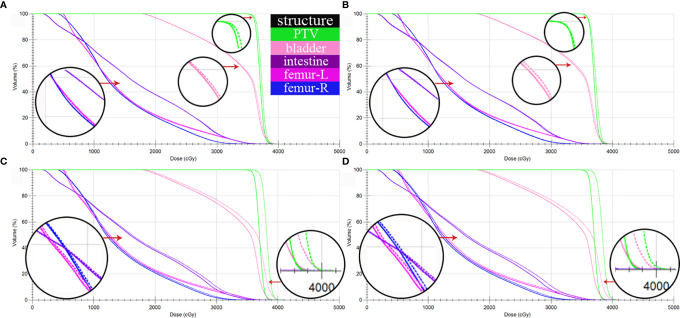
DVH comparison between CT_gt_ and pseudo CT obtained by three different deep learning methods. **(A)** DVH difference between CT_gt_ and CT_CycleGAN_. **(B)** DVH difference between CT_gt_ and CT_unet-GAN_. **(C)** DVH difference between CT_gt_ and CT_FCN-GAN_. **(D)** DVH difference between CTgt and CBCT. The solid line is the dose line of CT_gt_. The dotted line is the dose line of CT_gt_ or CBCT.

**Figure 10:**
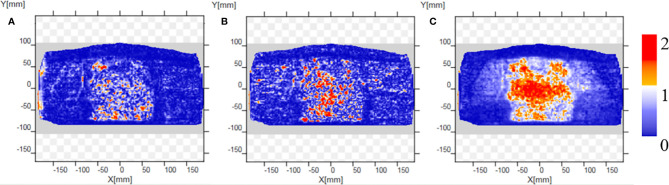
Comparison results of γ analysis (2%/2 mm) between the radiotherapy plan based on three types of pseudo-CT images and the radiotherapy plan based on CT_gt_ images. The bluer the dots in the difference map, the smaller the dose difference between the two plans, and the higher the overall γ-pass rate. **(A)** The pass rate graph of gamma analysis based on CT_CycleGAN._ **(B)** The pass rate graph of gamma analysis based on CT_unet-GAN._ **(C)** The pass rate graph of gamma analysis based on CT_FCN-GAN_.

CT_gt_ is compared with pseudo-CT images acquired through three methods in the DVH plot as shown in **Figure 9**, where the solid line is a DVH plot of a radiotherapy plan prepared based on patient CT_gt_ image, and the dotted line is a DVH plot based on a pseudo-CT image. **Figure 9A** shows a DVH difference plot between CT_CycleGAN_ and CT_gt_. **Figure 9B** displays a DVH difference plot between CT_unet-GAN_ and CT_gt_. **Figure 9C** is a DVH difference plot between CT_FCN-GAN_ and CT_gt_. The overlapping degree of volumetric dose curves of multiple OARs in the DVH plot between CT_CycleGAN_ and CT_gt_ was the highest, and the volumetric dose curve difference in PTVs of the two was also small. In comparison with CT_CycleGAN_, the volumetric dose curve of OARs in the DVH difference plot between CT_unet-GAN_ and CT_gt_ was different, the volumetric dose curve of PTV and multiple OARs of CT_FCN-GAN_ differed considerably from that of CT_gt_, and the volume of its high-dose region was also large. **Figure 9D** shows the difference of DVH between CBCT and CT_gt_. **Table 4** lists the comparison results of dose indexes in PTV and OARs in the radiotherapy plan based on four CT images and one CBCT image. The average, maximum, and minimum doses in OARs and PTV in the CT_CycleGAN_-based radiotherapy plan differed minimally from those in CT_gt_-based radiotherapy plans. **Figure 10** shows the comparison result of γ analysis (2%/2 mm) between the radiotherapy plan based on three types of pseudo-CT images and the radiotherapy plan based on CT_gt_ images. We use 90% γ-pass rate as the standard. The bluer the dots in the difference map, the smaller the dose difference between the two plans, and the higher the overall γ-pass rate. The three plans based on CT_CycleGAN_, CT_unet-GAN_, and CT_FCN-GAN_ had a γ-pass rate of 97.0%, 93.7%, and 84.9% respectively, indicating that the dose difference between the plans based on CT_CycleGAN_ and CT_gt_ was the smallest.

**Table 4:** Comparison of dose indexes of PTV and OARs in four CT images based on Monte Carlo optimization.

Plan Name	Metric	PTV	intestine	bladder	Femur-L	Femur-R
**CT_gt_**	Dmax	N/A	3,742.0	3,930.8	3,666.9	3,440.6
	Dmin	N/A	157.5	1749.3	478.1	406.7
	Dmean	3,701.4	1,681.2	3,281.5	1,451.1	1,401.3
	D98%	3,583.7	N/A	N/A	N/A	N/A
	D2%	3,819.1	N/A	N/A	N/A	N/A
**CT_CycleGAN_**	Dmax	N/A	3,746.9	3,938.2	3,683.9	3,453.0
	Dmin	N/A	148.4	1,737.8	485.1	403.7
	Dmean	3,721.1	1,678.6	3,296.1	1,438.3	1,407.3
	D98%	3595.4	N/A	N/A	N/A	N/A
	D2%	3828.3	N/A	N/A	N/A	N/A
**CT_unet-GAN_**	Dmax	N/A	3,753.4	4,001.5	3,758.6	3,455.3
	Dmean	3,726.8	1,681.3	3,301.0	1,444.0	1,396.1
	Dmin	N/A	143.0	1,729.1	464.2	404.1
	Dmean	3,726.8	1,681.3	3,301.0	1,444.0	1,396.1
	D98%	3,601.7	N/A	N/A	N/A	N/A
	D2%	3,857.7	N/A	N/A	N/A	N/A
**CT_FCN-GAN_**	Dmax	N/A	3816.3	4053.0	3792.0	3544.3
	Dmin	N/A	153.7	1775.9	501.8	417.9
	Dmean	3,818.5	1,713.8	3,374.0	1,484.7	1,428.1
	D98%	3,684.1	N/A	N/A	N/A	N/A
	D2%	3943.9	N/A	N/A	N/A	N/A
**CBCT**	Dmax	N/A	3,861.9	4,056.5	3,840.2	3,604.8
	Dmin	N/A	162.6	1,808.1	506.7	417.0
	Dmean	3,837.3	1,742.7	3,378.7	1,505.2	1,447.1
	D98%	3,687.6	N/A	N/A	N/A	N/A
	D2%	3,974.0	N/A	N/A	N/A	N/A

Dmax, maximum dose; Dmean, mean dose; Dmin, minimum dose; D98%, dose covering 98% of the PTV; D2%, dose to 2% of the PTV.

## Discussion

Pseudo-CT images acquired through deep learning methods based on CBCT images have various advantages in clinical radiotherapy and can solve the poor CBCT imaging quality of soft tissues and the impossible direct correction of an adaptive radiotherapy plan. In clinical ART, a CT_gt_ image applied to plan correction is acquired based on an image registration algorithm. This method needs to set the corresponding objective function according to the complexity of the registration region or anatomical structure. The non-rigid registration method is time-consuming for image registration tasks with complex texture information. Given that the boundary between the soft tissues of the CT(CBCT) image is not clear, the registration accuracy is not accurate. The CycleGAN-based deep learning method can construct the nonlinear mapping relationship between two image domains by a multilayer convolutional neural network with high feature extraction effect and efficiency, so it can solve the disadvantages of the deformation registration method.

According to comparative experimental results in the aspects of anatomical structure and dosimetry, the FCN-GAN-based method has unsatisfactory results in the skeleton region of synthetic rigid structure and the soft tissue region of nonrigid structure because paired data after registration are needed in FCN-GAN training. Otherwise, data mismatching and distortion can be easily caused. Moreover, the generator based on a fully convolutional neural network only contains convolutional layers without a shortcut connection of residual network, so it fails to combine superficial-layer features with deep-layer features. Consequently, the accuracy of model synthesis is degraded. The Unet-GAN-based method has a better synthetic effect than the former, but partial deformation still takes place in the skeleton region and some soft tissues are also inaccurate because preprocessed paired volume data are also needed for its training. A pooling layer is included in the U-Net network, resulting in losses of feature information of some anatomical structures. Then, the HU value in the synthesized pseudo-CT image is inaccurate. The improved CycleGAN network method proposed in this study can acquire pseudo-CT images better. Compared with a conventional GAN network only containing an adversarial loss function, the CycleGAN carrying cycle loss is a GAN synthetic network that contains two symmetrical mapping relations (CBCT→CT and CT→CBCT). The cycle loss function is L2 Euclidean distance between the input of the original image domain and pseudo image output in the same image domain acquired by twice feature transformation. Based on this bidirectional feature transformation pattern, the model can be trained without needing paired data (34). Owing to the existence of deformation and positioning set up errors of soft tissues to different degrees, the image data of the same patient acquired in different periods during the clinical radiotherapy are not the same, but the CycleGAN network can train unpaired CBCT-CT volume data to acquire pseudo image data, so it conforms to clinical practical application. However, 3D volume data are rich in feature information, so guaranteeing the accuracy of texture feature information of synthesized images only through the traditional loss function. Thus, the 3D gradient loss function was added into the objective function in this study to reserve detailed information of pseudo-CT images as much as possible.

An encoding–decoding pattern with a residual connection is used for the generator of the 3D CycleGAN network. Under this pattern, the output sharing the same dimensionality with the input is acquired through down-sampling, feature transformation, and up-sampling operations of the input volume data. Redundant feature information in the image can be compressed, to effectively extract image feature information (35). This network will splice the output of the previous layer with the output of the most adjacent convolution block before each up-sampling layer. This residual connection mode ensures integrating features of different layers during pseudo-CT synthesis. When increasing the network depth, this mode can improve the utilization efficiency of volume data features, acquiring accurate pseudo-CT images. In addition, if the network is operated by using 3D volume data of global dimensions as the input, considerable GPU video memory will be consumed, so parallel input mode based on image block can help to extract additional local feature information of the image while saving training time and reducing video memory consumption (36).

All data adopted in this study were volume data of abdominal cervical cancer patients. Relative to head and neck images, abdominal images acquired in different periods could have evident deformation changes in their internal soft tissues and OARs. The accuracy and reliability of images generated based on the CycleGAN could be better verified. Furthermore, the 3D pseudo-CT image acquired based on a 3D training model had spatial information in the Z direction, and it could not only be applied to positioning verification but also to the correction of radiotherapy plans. The pseudo-CT images synthesized by 2D CycleGAN cannot guarantee the continuity of the Z direction. Each layer is synthesized independently, leading to false registration between the images in two image domains in the 2D image synthesis task, resulting in false pseudo-CT synthesis. For example, establishing a mapping relationship between one CBCT slice and multiple CT slices is possible. If the pseudo-CT images synthesized based on 2D CycleGAN are not subjected to image post-processing, the final reconstructed 3D images cannot easily be used in clinical radiotherapy.

The pseudo-CT acquisition method based on 3D CycleGAN also has limitations. Given that the network contains GAN networks in two synthetic directions, the training speed is lower than that of unidirectional GAN. A pseudo-CT image with a satisfactory effect can be acquired only by multiple epochs. In the subsequent experiment, patch image blocks of different input dimensions will be adjusted by debugging hyperparameters of generators and discriminators, like learning rate, to elevate the training speed. In addition, multiple regions of interest will be divided according to density differences of OARs and different objective functions will be set to realize stepwise pseudo-CT synthesis, thereby further improving imaging quality of pseudo-CT images in the aspect of local details.

## Conclusion

An improved method of acquiring pseudo-CT images based on a 3D CycleGAN network with residual connections and attention gates was raised in this study. In the aspect of anatomical structure verification, the similarity degree of texture greyscale information of pseudo-CT images obtained through the new method with that of CT_gt_ images was experimentally proven higher in comparison with other GAN deep learning methods. For the sake of dosimetry verification, the dose distributions between radiotherapy plans prepared based on CT_gt_ image and those prepared based on pseudo-CT images acquired through the improved method were approximate under the same optimization conditions. Owing to its capability of eliminating the disadvantages of CBCT images in practical clinical application, the pseudo-CT image has outstanding application prospects in adaptive radiotherapy of cervical cancer.

## Data Availability Statement

The raw data supporting the conclusions of this article will be made available by the authors, without undue reservation.

## Ethics Statement

The studies involving human participants were reviewed and approved by the medical ethics committee of the Second People’s Hospital of Changzhou, Nanjing Medical University (2017-002-01). The patients/participants provided their written informed consent to participate in this study.

## Author Contributions

HS performed the experimental characterizations and analyzed the results. RF focused on the image segmentation and assisted to train the 3D CycleGAN. CL assisted to perform the experimental characterizations and worked out a radiotherapy plan. ZL collected the image data assisted to preprocess the images. KX assisted to collected the image data and evaluated the radiotherapy plan. XN conceived the experiments and revised the manuscript. JY revised the manuscript. All authors contributed to the article and approved the submitted version.

## Funding

This work was supported in part by the General Program of Jiangsu Provincial Health Commission (No. M2020006), in part by the Changzhou Key Laboratory of Medical Physics (No. CM20193005), and in part by the Innovation Foundation for Doctor Dissertation of Northwestern Polytechnical University (No. CX202039).

## Conflict of Interest

The authors declare that the research was conducted in the absence of any commercial or financial relationships that could be construed as a potential conflict of interest.

## References

[1] Eichenauer DA Aleman BMP André M Federico M Hutchings M Illidge T. Hodgkin lymphoma: ESMO Clinical Practice Guidelines for diagnosis, treatment and follow-up. Ann Oncol (2018) 29:19–29. 10.1093/annonc/mdy080 29796651

[2] Toita T Ariga T Kasuya G Hashimoto S Maemoto H Heianna J. Current Status and Perspective of Chemoradiotherapy for Uterine Cervical Cancer. Gan to kagaku ryoho. Cancer Chemother (2015) 42(10):1156–61. 26489545

[3] Rodriguez N Algara M Foro P Lacruz M Reig A Membrive I. Predictors of Acute Esophagitis in Lung Cancer Patients Treated With Concurrent Three-Dimensional Conformal Radiotherapy and Chemotherapy. Int J Radiat Oncol Biol Phys (2009) 73(3):810–7. 10.1016/j.ijrobp.2008.04.064 18755556

[4] Quint S Hoogeman MS Ahmad R Dhawtal G Bondar L De Pree I. IGRT in EBRT for cervical cancer, a plan of the day strategy. Radiother Oncol (2009) 92:S100–1. 10.1016/S0167-8140(12)72856-6

[5] Liu C Kumarasiri A Chetty IJ Kim J. SU-E-J-206: Delivered Dose to Organs From CBCT-Based IGRT of the Prostate. Med Phys (2013) 40(6):199–200. 10.1118/1.4814418

[6] Meroni S Mongioj V Giandini T Bonfantini F Cavallo A Carrara M. EP-1822: limits and potentialities of the use of CBCT for dose calculation in adaptive radiotherapy. Radiother Oncol (2016) 119:S854–5. 10.1016/S0167-8140(16)33073-0

[7] Siewerdsen JH Moseley DJ Bakhtiar B Richard S Jaffray DA. The influence of antiscatter grids on soft-tissue detectability in cone-beam computed tomography with flat-panel detectors. Med Phys (2004) 31(12):3506–20. 10.1118/1.1819789 15651634

[8] Mail N Moseley DJ Siewerdsen JH Jaffray DA. The influence of bowtie filtration on cone-beam CT image quality. Med Phys (2009) 36(1):22–32. 10.1118/1.3017470 19235370

[9] Kyriakou Y Riedel T Kalender WA. Combining deterministic and Monte Carlo calculations for fast estimation of scatter intensities in CT. Phys Med Biol (2006) 51(18):4567–86. 10.1088/0031-9155/51/18/008 16953043

[10] Yang X Wu S Sechopoulos I Fei B. Cupping artifact correction and automated classification for high-resolution dedicated breast CT images. Med Phys (2012) 39(10):6397–406. 10.1118/1.4754654 PMC3477198 23039675

[11] Qu X Lai CJ Zhong Y Yi Y Shaw CC. A general method for cupping artifact correction of cone-beam breast computed tomography images. Comput Assisted Radiol Surg (2016) 11(7):1233–46. 10.1007/s11548-015-1317-8 26514684

[12] Usui K Inoue T Kurokawa C Sugimoto S Ogawa K. SU-F-J-70: Monte Carlo Study On a Cone-Beam Computed Tomography Using a Cross-Type Carbon Fiber Antiscatter Grid. Med Phys (2016) 43(6):3422–5. 10.1118/1.4955978

[13] Chevillard C Dumas JL Mazal A Husson F. 42. Computation of the RT dose of the day from mapping CBCT information to the planning CT using an optimized elastic registration method. Physica Med (2017) 44:20–1. 10.1016/j.ejmp.2017.10.067

[14] Derksen A Koenig L Meine H Heldmann S. SU-F-J-97: A Joint Registration and Segmentation Approach for Large Bladder Deformations in Adaptive Radiotherapy. Med Phys (2016) 43(6):3429–9. 10.1118/1.4956005

[15] Abe T Tateoka K Saito Y Nakazawa T Yano M Nakata K. Method for Converting Cone-Beam CT Values into Hounsfield Units for Radiation Treatment Planning. Int J Med Phys Clin Eng Radiat Oncol (2017) 06(4):361–75. 10.4236/ijmpcero.2017.64032

[16] Lei Y Tang X Higgins KA Lin J Jeong J Liu T. Learning-based CBCT correction using alternating random forest based on auto-context model. Med Phys (2018) 46(2):601–18. 10.1002/mp.13295 PMC7792987 30471129

[17] Wang H Barbee D Wang W Pennell R Osterman K. SU-F-J-109: Generate Synthetic CT From Cone Beam CT for CBCT-Based Dose Calculation. Med Phys (2016) 43(6Part10):3432–2. 10.1118/1.4956017

[18] Zhu JY Park T Isola P Efros AA. Unpaired image-to-image translation using cycle-consistent adversarial networks. In: Proceedings of the IEEE international conference on computer vision. Venice: IEEE (2017). p. 2223–32. 10.1109/ICCV.2017.244

[19] Liang X Chen L Nguyen D Zhou Z Gu X Yang M. Generating synthesized computed tomography (CT) from cone-beam computed tomography (CBCT) using CycleGAN for adaptive radiation therapy. Phys Med Biol (2019) 64(12):125002. 10.1088/1361-6560/ab22f9 31108465

[20] Kida S Kaji S Nawa K Imae T Nakamoto T Ozaki S. Visual enhancement of Cone-beam CT by use of CycleGAN. Med Phys (2020) 47(3):998–1010. 10.1002/mp.13963 31840269

[21] Chen H Dou Q Yu L Qin J Heng PA. VoxResNet: Deep voxelwise residual networks for brain segmentation from 3D MR images. Neuroimage (2017) 170:S1053811917303348. 10.1016/j.neuroimage.2017.04.041 28445774

[22] Qiao Z Cui Z Niu X Geng S Yu Q. Image Segmentation with Pyramid Dilated Convolution Based on ResNet and U-Net. In: International Conference on Neural Information Processing. Cham: Springer (2017).

[23] Kudo Y Aoki Y. Dilated convolutions for image classification and object localization. In: 2017 Fifteenth IAPR International Conference on Machine Vision Applications (MVA). Nagoya: IEEE (2017). p. 452–5. 10.23919/MVA.2017.7986898

[24] He K Zhang X Ren S Jian S. Deep residual learning for image recognition. Comput Vision Pattern Recog (2016) 770–8. 10.1109/CVPR.2016.90

[25] Liu Y Lei Y Wang T Fu Y Tang X Curran WJ. CBCT-based Synthetic CT Generation using Deep-attention CycleGAN for Pancreatic Adaptive Radiotherapy. Med Phys (2020) 47(6):2472–83. 10.1002/mp.14121 PMC7762616 32141618

[26] Zhao J Zhang J Li Z Hwang JN Gao Y Fang Z. DD-CycleGAN: Unpaired image dehazing via Double-Discriminator Cycle-Consistent Generative Adversarial Network. Eng Appl Artif Intell (2019) 82(JUN.):263–71. 10.1016/j.engappai.2019.04.003

[27] Zhou Z Wang Y Guo Y Qi Y Yu J. Image Quality Improvement of Hand-Held Ultrasound Devices With a Two-Stage Generative Adversarial Network. IEEE Trans Biomed Eng (2020) 67(1):298–311. 10.1109/TBME.2019.2912986 31021759

[28] Nie D Trullo R Lian J Petitjean C Ruan S Wang Q. Medical Image Synthesis with Context-Aware Generative Adversarial Networks. In: Medical image computing and computer assisted intervention. Cham: Springer (2017). p. 417–25. 10.1007/978-3-319-66179-7_48 PMC6044459 30009283

[29] Lee J Nishikawa R Reiser I Boone J. WE-G-207-05: Relationship Between CT Image Quality, Segmentation Performance, and Quantitative Image Feature Analysis. Med Phys (2015) 42(6):3697–7. 10.1118/1.4926098

[30] He K Cao X Shi Y Nie D Gao Y Shen D. Pelvic Organ Segmentation Using Distinctive Curve Guided Fully Convolutional Networks. IEEE Trans Med Imaging (2019) 38(2):585–95. 10.1109/TMI.2018.2867837 PMC6392049 30176583

[31] Nithiananthan S Schafer S Mirota DJ Stayman JW Zbijewski W Reh DD. Extra-dimensional Demons: A method for incorporating missing tissue in deformable image registration. Med Phys (2012) 39(9):5718–31. 10.1118/1.4747270 PMC3443194 22957637

[32] Hore A Ziou D. Image quality metrics: PSNR vs. SSIM//2010 20th International Conference on Pattern Recognition. IEEE (2010) 2366–9. 10.1109/ICPR.2010.579

[33] Wendling M Zijp LJ Mcdermott LN Smit EJ Sonke JJ Mijnheer BJ. A fast algorithm for gamma evaluation in 3D. Med Pehysics (2007) 34(5):1647–54. 10.1118/1.2721657 17555246

[34] Almahairi A Rajeswar S Sordoni A Bachman P Courville A. Augmented CycleGAN: Learning Many-to-Many Mappings from Unpaired Data. Int Conf Mach Learn (2018) 195–204.

[35] Chen H Zhang Y Kalra MK Lin F Chen Y Liao P. Low-Dose CT With a Residual Encoder-Decoder Convolutional Neural Network. IEEE Trans Med Imaging (2017) 36(12):2524–35. 10.1109/TMI.2017.2715284 PMC5727581 28622671

[36] Long J Shelhamer E Darrell T. Fully Convolutional Networks for Semantic Segmentation. IEEE Trans Pattern Anal Mach Intell (2015) 39(4):640–51. 10.1109/TPAMI.2016.2572683 27244717

